# Supplementary material for: Inositol pyrophosphate dynamics reveals control of the yeast phosphate starvation program through 1,5-IP8 and the SPX domain of Pho81
Source: eLife. 2023 Sep 20;12:RP87956. doi: 10.7554/eLife.87956 (PMC10511240; doi:10.7554/eLife.87956)
Supplement: Supplementary file 1. — (a) List of strains. (b) List of plasmids. (c) List of primers used for genetic manipulations. [file elife-87956-supp1.docx]

**Appendix**

# Supplementary file 1a: Strains used in this study

The *S. cerevisiae* strains used in this study were all obtained by genetic manipulations from the BY4741 background described in Table S1. This strain corresponds to the wild-type of this study. For the sake of clarity, the BY4741 background was therefore not indicated in the genotype of each mutant strain.

| Strain | Genotype | Plasmid | Source |
| --- | --- | --- | --- |
| Wild-type (BY4741) | *MATa his3Δ1 leu2Δ0 met15Δ0 ura3Δ0* |  | Euroscarf |
| *pho4-yEGFP* | *pho4-link-yEGFP-CaURA3 hta2-mCherry*^a^ |  | This study |
| *pho81-yEGFP* | *pho81-link-yEGFP-CaURA3* |  | This study |
| *pho81Δspx* | *pho81Δ200*^a^ |  | This study |
| *pho81Δspx/pho4-yEGFP* | *pho81Δ200*^a^ *pho4-link-yEGFP-CaURA3 hta2-mCherry*^a^ |  | This study |
| *pho81Δspx-yEGFP* | *pho81Δ200*^a^ *pho81-link-yEGFP-CaURA3* |  | This study |
| *pho81Δspx/prPho5-yEGFP* | *pho81Δ200*^a^ | pVC115 | This study |
| *vip1Δ* | *vip1Δ0*^a^ |  | This study |
| *vip1Δ/pho4-yEGFP* | *vip1Δ0*^a^ *pho4-link-yEGFP-CaURA3 hta2-mCherry*^a^ |  | This study |
| *vip1Δ/pho81-yEGFP* | *vip1Δ0*^a^ *pho81-link-yEGFP-CaURA3* |  | This study |
| *vip1Δ/prPho5-yEGFP* | *vip1Δ0*^a^ | pVC115 | This study |
| *kcs1Δ* | *kcs1Δ0::kanMX4* |  | ^1^ |
| *kcs1Δ/pho4-yEGFP* | *kcs1Δ0::kanMX4 pho4-link-yEGFP-CaURA3 hta2-mCherry*^a^ |  | This study |
| *kcs1Δ/pho81-yEGFP* | *kcs1Δ0::kanMX4 pho81-link-yEGFP-CaURA3* |  | This study |
| *kcs1Δ/prPho5-yEGFP* | *kcs1Δ0::kanMX4* | pVC115 | This study |
| *vip1Δ/kcs1Δ* | *vip1Δ0*^a^ *kcs1Δ0::kanMX4* |  | This study |
| *vip1Δ/kcs1Δ/pho4-yEGFP* | *vip1Δ0*^a^ *kcs1Δ0::kanMX4 pho4-link-yEGFP-CaURA3*  *hta2-mCherry*^a^ |  | This study |
| *vip1Δ/kcs1Δ/pho81-yEGFP* | *vip1Δ0*^a^ *kcs1Δ0::kanMX4 pho81-link-yEGFP-CaURA3* |  | This study |
| *vip1Δ/kcs1Δ/prPho5-yEGFP* | *vip1Δ0*^a^ *kcs1Δ0::kanMX4* | pVC115 | This study |
| *prGPD-vip1/kcs1Δ* | *kcs1Δ0::kanMX4* | pVC97 | This study |
| *prGPD-vip1/kcs1Δ/pho4-yEGFP* | *kcs1Δ0::kanMX4 pho4-link-yEGFP-CaURA3 hta2-mCherry*^a^ | pVC97 | This study |
| *prGPD-vip1/kcs1Δ/pho81-yEGFP* | *kcs1Δ0::kanMX4 pho81-link-yEGFP-CaURA3* | pVC97 | This study |
| *prGPD-vip1/kcs1Δ/prPho5-yEGFP* | *kcs1Δ0::kanMX4* | pVC115, pVC114 | This study |
| *pho81^K154A^* | *pho81^K154A a^* |  | This study |
| *pho81^K154A^/pho4-yEGFP* | *pho81^K154A a^ pho4-link-yEGFP-CaURA3 hta2-mCherry*^a^ |  | This study |
| *pho81^K154A^/pho81-yEGFP* | *pho81^K154A^-link-yEGFP-CaURA3^a^* |  | This study |
| *pho81^K154A^/prPho5-yEGFP* | *pho81^K154A a^* | pVC115 | This study |
| *pho81^K154A^/vip1Δ/pho4-yEGFP* | *pho81^K154A a^ vip1Δ0*^a^ *pho4-link-yEGFP-CaURA3 hta2-mCherry*^a^ |  | This study |
| *pho80^R121K^/pho81-yEGFP* | *pho80^R121K a^ pho81-link-yEGFP-CaURA3* |  | This study |
| *pho80^R121K^/kcs1Δ/pho81-yEGFP* | *pho80^R121K a^ kcs1Δ0::kanMX4 pho81-link-yEGFP-CaURA3* |  | This study |
| *pho80^R121K^/vip1Δ/pho81-yEGFP* | *pho80^R121K a^ vip1Δ0*^a^ *pho81-link-yEGFP-CaURA3* |  | This study |
| *pho80^R121K^/pho81^K154A^-yEGFP* | *pho80^R121K a^ pho81^K154A^-link-yEGFP-CaURA3^a^* |  | This study |
| *pho80^R121K^/pho81Δspx-yEGFP* | *pho80^R121K a^ pho81Δ200-link-yEGFP-CaURA3^a^* |  | This study |
| *pho80^E154V^/pho81-yEGFP* | *pho80^E154V a^ pho81-link-yEGFP-CaURA3* |  | This study |
| *pho80^E154V^/kcs1Δ/pho81-yEGFP* | *pho80^E154V a^ kcs1Δ0::kanMX4 pho81-link-yEGFP-CaURA3* |  | This study |
| *pho80^E154V^/vip1Δ/pho81-yEGFP* | *pho80^E154V a^ vip1Δ0*^a^ *pho81-link-yEGFP-CaURA3-hta2mCh* |  | This study |
| *pho80^E154V^/pho81^K154A^-yEGFP* | *pho80^E154V a^ pho81^K154A^-link-yEGFP-CaURA3^a^* |  | This study |
| *pho80^E154V^/pho81Δspx-yEGFP* | *pho80^E154V a^ pho81Δ200-link-yEGFP-CaURA3^a^* |  | This study |
| BY4742 | *MATa his3Δ1 leu2Δ0 lys2Δ0 ura3Δ0* |  | Euroscarf |
| *BY4742 pho80Δ* | *pho80Δ0::natNT2 (A3464)* |  | This study |

^a^ obtained by the CRISPR-Cas9 method

# Supplementary file 1b: Plasmids

| Plasmid | Description | Source |
| --- | --- | --- |
| pVC115 | pCEV-G1-LEU- *prPHO5-yegfp prTEF-mcherry leu2* | This study |
| pVC97 | pRS415 *prGPD-vip1 leu2* | This study |
| pVC114 | pRS413 *prGPD-vip1 his3* | This study |
| pED109 | *pGTL-mcherry* | Serge Pelet |
| pSP473 | pRS423 *prGPD-cas9 his3* | Serge Pelet |
| pSP475 | pRS425 *prGPD-cas9 leu2* | Serge Pelet |
| pSP476 | pRS426 *prGPD-cas9 ura3* | Serge Pelet |
| pSP478 | pRS425 *prGPD-cas9_sgRNA_hta2 leu2* | Serge Pelet |
| pVC24 | pRS425 *prGPD-cas9_sgRNA_pho81 leu2* | This study |
| pVC43 | pRS425 *prGPD-cas9_sgRNA_kcs1 leu2* | This study |
| pVC44 | pRS423 *prGPD-cas9_sgRNA_vip1 his3* | This study |
| pVC110 | pRS425 *prGPD-cas9_sgRNA_vip1 leu2* | This study |
| pVC72 | pRS423 *prGPD-cas9_sgRNA_pho80_1 his3* | This study |
| pVC73 | pRS423 *prGPD-cas9_sgRNA_pho80_2 his3* | This study |
| pVC108 | pRS425 *prGPD-cas9_sgRNA_pho80_1 leu2* | This study |
| pVC109 | pRS425 *prGPD-cas9_sgRNA_pho80_2 leu2* | This study |
| pVC119 | pRS315 *hta2-mCherry* | This study |
| pGK1 | pCEV-G1-LEU- *prPHO84-yegfp prTEF-mcherry leu2* | This study |
| pMJK1 | pRS416 *prADH1_Pho81-SPX^1-394^-yEGFP ura3* | This study |

# Supplementary file 1c: Oligonucleotides

| Oligonucleotide | Sequence |
| --- | --- |
| 645_fw | ACTTGTTGCCAAAGAAGTCTGCCAAGACTGCCAAAGCTTCTCAAGAACTGGCGGCCGCTCTAGAACTA |
| 1435_rev | TACTATACACTGTCTTTAATAAAACGACGCTATTTATAAATTATTTAGAAGTGGCGCGCC |
| CRISPR_sg_F | GGTCAAACGCTGTAGAAGTG |
| Pho81_K150A_F2 | TATGTTGAGTTAAATAAAACGGGATTTTCAGCAGCTCTGAAGAAATGGGACAAGAGATCTCAATCTCACGATAAAGATTTTTATCTTGCTACTGTTGTTTCCATTCAACCAATT |
| Pho81_K150A_R2 | AATTGGTTGAATGGAAACAACAGTAGCAAGATAAAAATCTTTATCGTGAGATTGAGATCTCTTGTCCCATTTCTTCAGAGCTGCTGAAAATCCCGTTTTATTTAACTCAACATA |
| Pho81_K154A_F2 | AATAAAACGGGATTTTCAAAAGCTCTGAAGGCATGGGACAAGAGATCTCAATCTCACGATAAAGATTTTTATCTTGCTACTGTTGTTTCCATTCAACCAATT |
| Pho81_K154A_R2 | AATTGGTTGAATGGAAACAACAGTAGCAAGATAAAAATCTTTATCGTGAGATTGAGATCTCTTGTCCCATGCCTTCAGAGCTTTTGAAAATCCCGTTTTATT |
| Pho81_sgRNA_F | TTCAAAAGCTCTGAAGAAATGTTTTAGAG |
| Pho81_sgRNA_R | CTAGCTCTAAAACATTTCTTCAGAGCTTTTGAAACGT |
| Vip1_sgRNA_F | TCGTAGCATATTAATATATTGCAGAAGGTCTGATCACACCCAATTTTTAATTTAGTAACC |
| Vip1_sgRNA_R | GGTTACTAAATTAAAAATTGGGTGTGATCAGACCTTCTGCAATATATTAATATGCTACGA |
| Kcs1_sgRNA_F | TTGTATATATAAAACTAAAGCTAAAAGACTAAAGAAAGGATAGAACTAATGAATATTCTT |
| Kcs1_sgRNA_R | AAGAATATTCATTAGTTCTATCCTTTCTTTAGTCTTTTAGCTTTAGTTTTATATATACAA |
| Pho80_sgRNA_F1 | TTAATTCGTTGACTGCCCATGTTTTAGAG |
| Pho80_sgRNA_R1 | CTAGCTCTAAAACATGGGCAGTCAACGAATTAAACGT |
| Pho80_sgRNA_F2 | ATGTCACGAATTGAATATACGTTTTAGAG |
| Pho80_sgRNA_R2 | CTAGCTCTAAAACGTATATTCAATTCGTGACATACGT |
| Pho80_R121K_F | ATTTTACGCTTAATTCGTTGACTGCCCATAAATTTTTATTAACAGCCACCACAGTCGCAA |
| Pho80_R121K_R | TTGCGACTGTGGTGGCTGTTAATAAAAATTTATGGGCAGTCAACGAATTAAGCGTAAAAT |
| Pho80_E154V_F | ATGCAAAAGTTGGAGGAGTACGATGTCACGTTTTGAATATACTTGAGAACGATTTTTTAAAGAGAGTAAACTAC |
| Pho80_E154V_R | GTAGTTTACTCTCTTTAAAAAATCGTTCTCAAGTATATTCAAAACGTGACATCGTACTCCTCCAACTTTTGCAT |
| Pho81_yEGFP_F | CTACGCTTGTGAGTTGCTTTTTGAGAATAATATTGATATGGGTGACGGTGCTGGTTTA |
| Pho81_yEGFP_R | TAATGTATAAGATTTCAAAACTACATATTACAGAACTTTATCGATGAATTCGAGCTCG |
| Pho81Δspx_F | GAGGCAACATAGATAGATAAACGTGCAATGGATATTGACAACAACAATAGAAGGGCAGAC |
| Pho81Δspx_R | GTCTGCCCTTCTATTGTTGTTGTCAATATCCATTGCACGTTTATCTATCTATGTTGCCTC |
| Vip1_F | GGGGATCCATGAGTGGGATAAAGAAGGAACC |
| Vip1_R | CCCGTCGACCTAATCTAATGTCTTGTTAACGG |
| Vip1_F2 | AATAACCTCACCGGTCACGG |
| Vip1_R2 | GTAAGACGTTATTTCCCACC |
| pCEV-G1_Leu_F1 | CGCCGGGTCACCCGGCCAGCCTGTGCGGTATTTCACACCG |
| pCEV-G1_Leu_R1 | CGGTGTGAAATACCGCACAGGCTGGCCGGGTGACCCGGCG |
| pCEV-G1_Leu_F2 | GTGCACTCTCAGTACAATCTCTGTCGATTCGATACTAACG |
| pCEV-G1_Leu_R2 | CGTTAGTATCGAATCGACAGAGATTGTACTGAGAGTGCAC |
| pPho5_-1500_F_Nae1 | TATGCCGGCCTACCAACATTTGGACCCGACAG |
| pPho5_-1_R_Xba1 | TGCTCTAGATGGTAATCTCGAATTTGCTTGCTCTATTTG |
| yegfp_F_Hind3 | CCCAAGCTTATGTCTAAAGGTGAAGAATTATTCACTGGTGT |
| yefgp_R_SacII | TCCCCGCGGTTATTTGTACAATTCATCCATACCATGGGTAATACC |
| mcherry_F_NotI | ATAAGAATGCGGCCGCATGGTTAGCAAAGGCGAGGAAGATAA |
| mcherry_R_PacI | ACCTTAATTAATTACTTGTACAGTTCATCCATACCACCTGTA |
| pPho84_P1F | CATTTTGAAGCTATGGTGTGTGCGGCCGGCAGAACAAAAATTTTGGCGTCGGGAGAGTAC |
| pPho84_P1R | GTACTCTCCCGACGCCAAAATTTTTGTTCTGCCGGCCGCACACACCATAGCTTCAAAATG |
| pPho84_P2F | AACAAACAAAACTCCACGAATACAATCCAATCTAGAACTAGTGGATCCCCCGGGCTGCAG |
| pPho84_P2R | CTGCAGCCCGGGGGATCCACTAGTTCTAGATTGGATTGTATTCGTGGAGTTTTGTTTGTT |
| Pho81_SPX-GFP_P1F | TTTCAAGCTATACCAAGCATACAATCAACTATGAAATTCGGCAAGTATTTGGAAGCCAGG |
| Pho81_SPX-GFP_P1R | CCTGGCTTCCAAATACTTGCCGAATTTCATAGTTGATTGTATGCTTGGTATAGCTTGAAA |
| Pho81_SPX-GFP_P2F | GGCTGCAAGTTGTCCAGAAAAATCAGGTGGTGGTGGTGGTTCAATGTCTAAAGGTGAAGAATTATTCACTGG |
| Pho81_SPX-GFP_P2R | CCAGTGAATAATTCTTCACCTTTAGACATTGAACCACCACCACCACCTGATTTTTCTGGACAACTTGCAGCC |
| Pho81_SPX-GFP_P3F | CCCATGGTATGGATGAATTGTACAAATAATCATGTAATTAGTTATGTCACGCTTACATTC |
| Pho81_SPX-GFP_P3R | GAATGTAAGCGTGACATAACTAATTACATGATTATTTGTACAATTCATCCATACCATGGG |
